# Toward a minimalist strategy for PFO closure: outcomes and patient experience from 205 ICE-guided procedures performed under local anesthesia and early discharge

**DOI:** 10.1007/s12928-026-01263-5

**Published:** 2026-03-04

**Authors:** Thibaut Pommier, Mathieu Mourot, Pierre Guilleminot, Charles Guenancia, Luc Lorgis

**Affiliations:** 1 Université Bourgogne Europe, CHU Dijon Bourgogne, Service de Cardiologie, PEC2 UR 7460, 21000 Dijon, France; 2 Université Bourgogne Europe, CHU Dijon Bourgogne, Service de Cardiologie, 21000 Dijon, France; 3 Hôpital Privé Dijon Bourgogne, Service de Cardiologie, 21000 Dijon, France; 4 Cardiology Department, University Hospital, 14 rue Paul Gaffarel, 21079 Dijon CEDEX, France

**Keywords:** Patent foramen ovale closure, Intracardiac echocardiography, Outpatient procedure, Local anesthesia, Minimalist interventional cardiology, Patient comfort

## Abstract

Patent foramen ovale (PFO) closure is an established treatment for secondary prevention after PFO-related stroke. Traditionally guided by transesophageal echocardiography (TEE) under general anesthesia, the procedure can now be performed using intracardiac echocardiography (ICE) under local anesthesia, supporting a minimalist and potentially outpatient approach. We retrospectively analyzed 205 consecutive patients who underwent ICE-guided PFO closure at Dijon University Hospital between 2018 and 2023. All procedures were performed under local anesthesia, without TEE or anesthesiology support. Procedural success, complications, discharge timing, and patient-reported comfort were evaluated, with clinical follow-up assessing recurrent events. Procedural success was achieved in 100% of patients, with a mean duration of 56 ± 19 min from catheterization laboratory entry to exit. Most patients (94%) were discharged within 24 h of admission, including 44% who underwent the procedure on an outpatient basis. Complications were infrequent (3.4% minor vascular events; one device embolization). Patient comfort was excellent (mean 9.1 ± 1.7/10). At follow-up, no recurrent ischemic events were observed. ICE-guided PFO closure under local anesthesia enables a simplified, anesthesia-free, and highly tolerated procedural pathway. These findings support the adoption of a minimalist interventional approach to streamline PFO management and optimize patient experience.

**Graphical Abstract:**

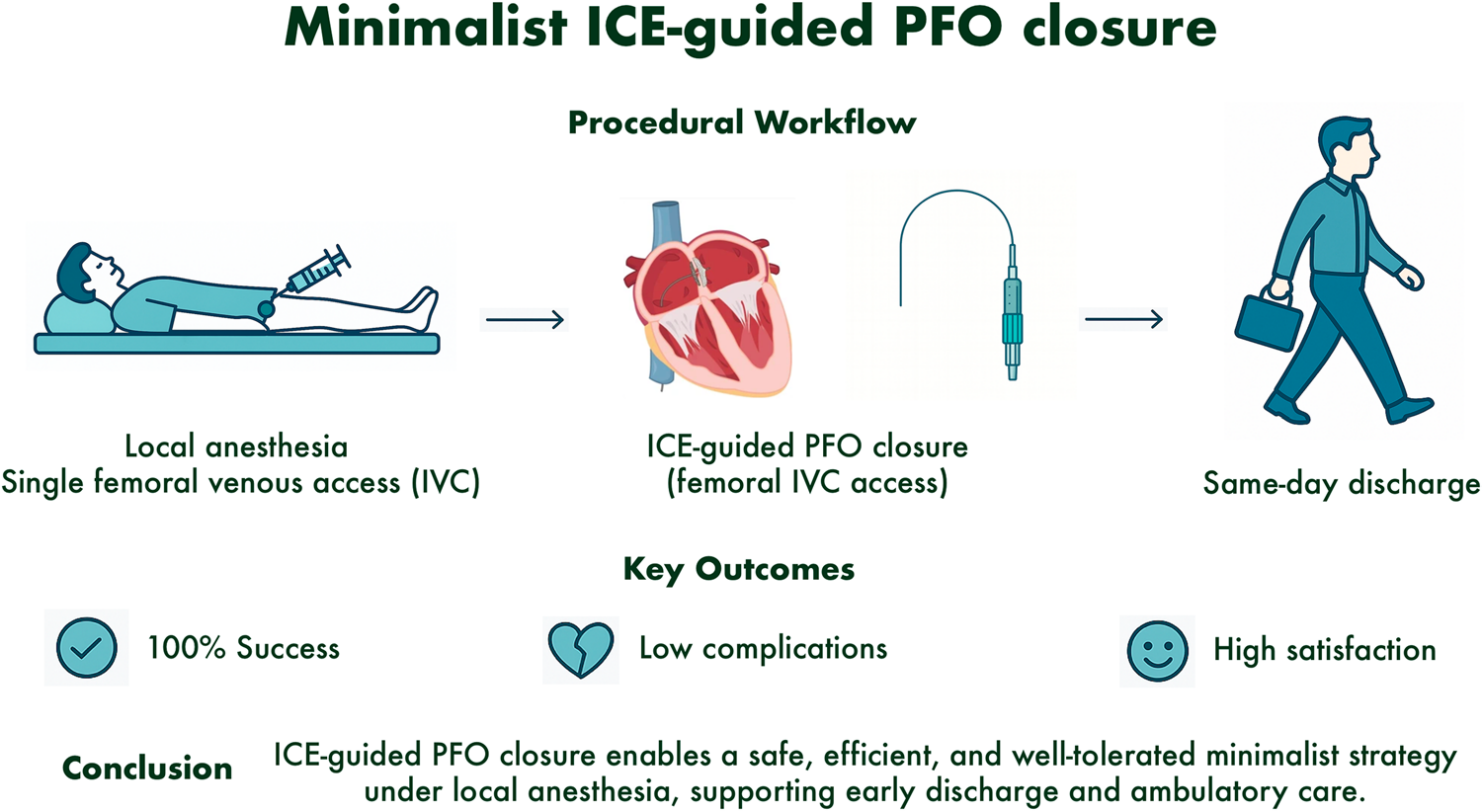

## Key learning points

**What is already known?**

- PFO closure is an established intervention for secondary prevention after PFO-related stroke.
- Traditional PFO closure is usually performed under general anesthesia with TEE guidance.
- Minimalist approaches using local anesthesia and ICE are increasingly explored to simplify the procedure and improve patient experience.

**What this study adds?**

- Demonstrates a minimalist ICE-guided PFO closure strategy, enabling same-day discharge or < 24 h hospitalization for the majority of patients.
- Confirms procedural safety and efficacy with 100% success and minimal complications with full ICE guidance.
- Short catheterization laboratory time, consistent with previously reported efficiencies of ICE-guided strategies.
- Enhances patient comfort by avoiding general anesthesia and TEE.

## Introduction

Patent foramen ovale (PFO) is a common congenital variant cardiac anatomy in approximately one-quarter of the population [1, 2] characterized by right-to-left interatrial blood shunting, due to an inconstant closure of foramen ovale after the birth. PFO is also implicated in the causation of stroke and could concern up to one half of patients with stroke of unknown cause [3–5]. In fact, the prevalence of PFO at autopsy range from 20 to 26% in the general population and may be as 56% in patients younger than 55 years old who have a cryptogenic stroke [6, 7].

The results of the RESPECT trial [3], the CLOSE trial [8], and the Gore-REDUCE trial [9] strongly demonstrated that PFO closure is superior to antiplatelet agents alone in reducing the recurrence rate of PFO-related stroke in patients under 60 years of age. Consequently, according to the 2024 European Stroke Organisation guidelines, PFO closure is recommended in patients aged 18–60 years with PFO-associated stroke, defined as ischemic stroke in the absence of any other identifiable cause except a PFO, in addition to antiplatelet therapy [10]. In fact, this population largely overlaps with a specific subgroup of patients classified as embolic stroke of undetermined source (ESUS), although ESUS represents a broader clinical category.

Traditionally, PFO closure has been performed under general anesthesia with transesophageal echocardiography (TEE) guidance, which remains the gold standard for device placement [11]. However, TEE-guided procedures entail several limitations, including patient discomfort, anesthesia-related risks, potential esophageal or tracheal injury, and the need for anesthesiology support. In contrast, intracardiac echocardiography (ICE) provides real-time, high-resolution imaging of the interatrial septum directly from the right atrium, enabling full procedural guidance under local anesthesia [12, 13].

Beyond its technical feasibility, ICE-guided intervention represents a shift toward a “minimalist” and patient-centered approach in structural heart disease. By eliminating the need for general anesthesia and TEE, ICE may shorten procedural times, simplify logistics, and facilitate outpatient management, improving both efficiency and patient experience. Meta-analyses have shown comparable procedural success and complication rates between ICE and TEE, with the additional advantages of shorter fluoroscopy time, reduced hospital stay, and improved comfort [14, 15].

At Dijon University Hospital, ICE-guided percutaneous intervention has been used for many years and today represents the reference guiding modality for PFO or even left atrial appendage closure [16]. Moreover, in our cardiology unit, ICE-guided PFO closures are performed on ambulatory care with an admission at hospital and an exit home the same day of the procedure (admission may be the day before if the patient prefers), after echocardiographic control confirming the absence of post procedural complications. This minimalist model aligns with current healthcare goals of resource optimization and patient-centered care, with a reduction of costs and intervention delays.

Thus, we sought to evaluate the feasibility, safety, and efficacy of a minimalist ICE–guided approach for PFO closure performed under local anesthesia, emphasizing its potential to streamline the procedure and enable same-day discharge without compromising clinical outcomes.

## Material and methods

### Study design and patient population

We conducted a retrospective study in all patients with PFO-related stroke underwent PFO closure between January 2018 and December 2023, in the interventional cardiology unit of the Dijon University Hospital.

We included patients fulfilling the European definition of PFO-related stroke, corresponding to a selected subgroup of ESUS patients in whom no alternative stroke etiology (no identifiable cause after excluding carotid or intracranial artery stenosis, atrial fibrillation, intracardiac thrombus, and atheromatous plaque at the aorta) was identified and PFO was considered as the most plausible mechanism.

PFO closure was subsequently validated by a multidisciplinary team comprising an interventional cardiologist, a cardiologist specialized in echocardiography, a neurologist, and a radiologist, based on clinical and echocardiographic data, as described below. Obviously, all patients had a PFO with closure criteria corresponding to the guidelines to previous studies with an associated atrial septal aneurysm (ASA) or large interatrial shunt.

### Preprocedural diagnostic work-up

All data were extracted from electronic medical records of the University Hospital of Dijon. The following baseline patient characteristics were collected: demographics (age, sex), cardiovascular risk factors (smoking, hypertension, overweight, diabetes, dyslipidemia), comorbidities (previous stroke, MI, deep vein thrombosis or pulmonary embolism, migraine), and medications such as estrogen-progestin contraceptives. We also collected data on the type of qualifying event (stroke or transient ischemic attack (TIA)), NIH Stroke Scale Score (NIHSS), RoPE score and CHA₂DS₂-VASc (calculated before the stroke event) score.

Screening for PFO was systematically performed as part of the standardized etiological work-up of ischemic stroke, particularly in patients younger than 60 years. Initial evaluation relied on transthoracic echocardiography (TTE) with contrast (agitated saline), including a Valsalva maneuver when feasible. In cases of suboptimal acoustic window or when a more detailed anatomical characterization was required, TEE was performed. PFO morphology and complexity were assessed based on established echocardiographic criteria, including the length and width of the PFO tunnel, the presence of an ASA or associated atrial septal defect (ASD), the thickness of the septum secundum, and associated anatomical features such as enlargement of the ascending aorta. In accordance with the CLOSE trial criteria [8], shunt severity was assessed by visual counting of microbubbles on contrast echocardiography and was considered significant when more than 30 microbubbles appeared in the left atrium within three cardiac cycles after opacification of the right atrium, whether the shunt was spontaneous or induced by a Valsalva maneuver, in accordance with previous studies. Atrial septal aneurysm was diagnosed on the basis of a septum primum excursion greater than 10 mm. Others standard echocardiographic parameters were collected, including left ventricular ejection fraction, left atrial volume index or potential valvulopathy. However, transcranial Doppler was not routinely used in our diagnostic pathway.

### Procedural workflow and procedure description

Percutaneous PFO closure were performed in Philips® cath lab and ICE imaging was performed using the 9-F ViewFlex® Xtra ICE catheter (Abbott) with the Zonare ViewMate® Ultrasound Console (Abbott).

The Amplatzer® PFO Occluder (Abbott), GORE® Septal Occluder (Gore Medical and Associates Inc., Newark, DE, USA), and CARDIA Ultrasep® PFO Occluder (Cardia) were the potential devices implanted in all patients.

All ICE-guided PFO closure procedures were performed under local anesthesia. Sometimes, slight sedation by benzodiazepines, hypnosis or virtual reality headset has been used to improve patient comfort. The procedures were performed by two experienced interventional cardiologists, one for deploying the device and the other for handling ICE.

The right and sometimes left femoral veins were punctured to introduce sheaths for the PFO closure device and ICE catheter. Most of the procedures in our center have been realized with a single venous access to limit the number of blood punctures, using the "Two-In-One Technique", minimizing vascular punctures and facilitating early discharge, as previously described [17]. Briefly, this venous access strategy involves ultrasound-guided puncture of the common femoral vein, followed by a small skin incision and pre-closure using a suture-based vascular closure device. An initial introducer sheath is inserted, through which two 0.035-inch guidewires are advanced. The sheath is then removed, allowing placement of the required introducer sheaths over each guidewire, typically a 10-F sheath for the ICE catheter and an 8- or 9-F sheath for the PFO delivery system. In cases of pre-closure failure, an additional suture-based closure device or manual compression was used to achieve hemostasis. After femoral venous access was obtained using the selected technique, intravenous heparin was administered to achieve an activated clotting time greater than 250 s, in accordance with current recommendations.

ICE setup and catheter advancement into the right atrium were systematically performed before introduction of the PFO delivery sheath, allowing early optimization of image quality and definition of a stable septal reference plane. The ICE catheter was positioned in the mid right atrium (under fluoroscopic guidance) with slight posterior flexion and clockwise rotation to obtain the optimal “PFO view,” enabling detailed visualization of the interatrial septum and PFO anatomy. Particular attention was paid to septal elongation during imaging in order to avoid underestimation of anatomical dimensions (for the sizing of the device).

Under ICE and fluoroscopy guidance, the PFO was then crossed using a 0.035-inch guidewire and a multipurpose catheter, with the guidewire advanced into the left upper pulmonary vein to provide stable support. The delivery sheath was subsequently advanced over the guidewire in parallel to the ICE catheter.

**Fig. 1:**
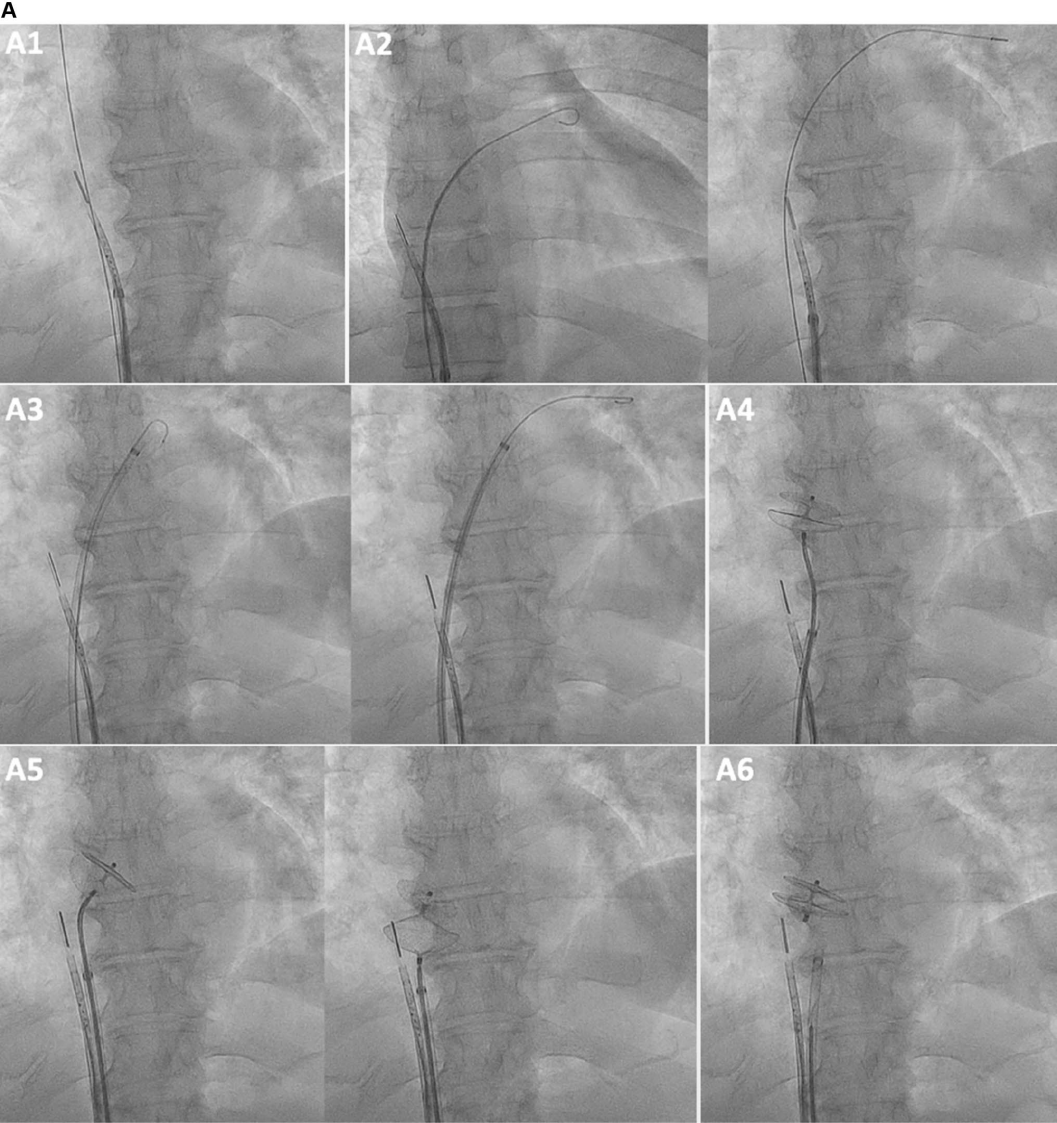

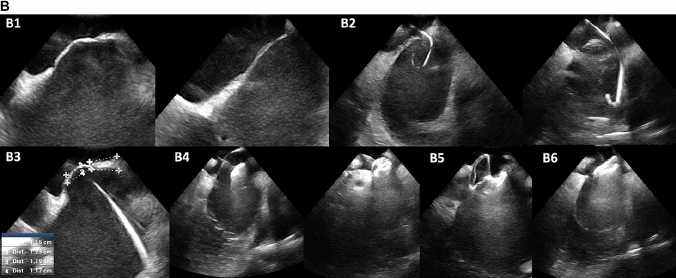
Representative angiographic and intracardiac echocardiography views illustrating the step-by-step workflow of minimalist ICE-guided PFO closure. **Panel A.** Angiographic views. **A1.** Initial angiographic position of the ICE catheter in the right atrium, serving as a reference for subsequent procedural steps. **A2.** Fluoroscopic visualization of PFO crossing using a multipurpose catheter and a 0.035-inch guidewire, with advancement and stabilization of the guidewire into the left upper pulmonary vein. **A3.** Positioning of the delivery sheath across the interatrial septum prior to device advancement, with guidewire withdrawal testing to confirm appropriate left atrial positioning under ICE guidance. **A4.** Device deployment under fluoroscopy, performed by gradual sheath retraction to allow controlled release of the device. **A5.** Stability assessment using standardized push-and-pull maneuvers to evaluate device anchoring and mobility before final detachment. **A6.** Final angiographic appearance after complete device release, confirming stable device position across the interatrial septum. **Panel B.** Intracardiac echocardiography views. **B1.** Initial ICE visualization of the patent foramen ovale, providing detailed assessment of interatrial septal anatomy and baseline PFO morphology. **B2.** ICE-guided visualization of guidewire passage across the PFO, confirming correct traversal of the interatrial septum. **B3.** Intraprocedural ICE-based anatomical measurements for device sizing, including assessment of PFO tunnel length, septal thickness, and the presence of atrial septal aneurysm. **B4.** ICE-guided device deployment with visualization of left atrial disc release and subsequent septal capture. **B5.** ICE confirmation of device stability during push-and-pull maneuvers, ensuring appropriate disc alignment and anchoring on both sides of the septum. **B6.** Final ICE assessment after device release, demonstrating correct device position, adequate septal apposition, and absence of immediate complications

Device deployment was performed predominantly under ICE guidance. The left atrial disc was first released and positioned against the septum, followed by gentle traction under continuous ICE visualization to ensure appropriate septal capture before releasing the right atrial disc. Device stability was assessed using standardized push-and-pull maneuvers, with ICE confirmation of correct disc alignment on both sides of the septum prior to final release. ICE was also used to verify final device position and exclude immediate complications. Before final release, all tension on the delivery system was systematically released to allow assessment of the device’s final resting position and stability under physiological conditions. At the end of the procedure, the catheter is removed and vascular closure was achieved using a suture-based closure device (Perclose ProGlide®, Abbott Cardiovascular) or manual compression. The step-by-step procedural workflow is illustrated in Fig. 1, combining representative angiographic and ICE views.

### Device selection and sizing

Device size selection was primarily guided by real-time intraprocedural ICE, which provided direct visualization of the interatrial septum and allowed dynamic assessment of PFO anatomy, enabling individualized device sizing rather than relying solely on preprocedural measurements. The choice of device size was based on a combination of anatomical criteria, including the length and width of the PFO tunnel, the presence and extent of an ASA, the thickness and width of the septum secundum, the presence of potential multiple septal fenestrations, and septal mobility. During the procedure, ICE was used to simulate device positioning and to assess disc alignment, septal capture, and stability prior to final release. The selected device aimed to ensure optimal anchoring, procedural safety, and the absence of significant residual interatrial shunt.

### Practical considerations and radiation minimization

ICE played a central role throughout the procedure, allowing device deployment with minimal reliance on fluoroscopy. Fluoroscopy was primarily and only used for catheter advancement, guidewire manipulation, and final confirmation of device stability prior to detachment. Several practical points were identified during adoption of this minimalist strategy. Adequate septal elongation during ICE imaging was systematically sought to avoid underestimation of PFO dimensions. Excessive device mobility after deployment was considered suggestive of insufficient anchoring and prompted reconsideration of device size. Device recapture and repositioning were safely performed under combined ICE and fluoroscopic guidance when needed, using controlled push-and-pull maneuvers and systematic release of tension to assess final device position. Radiation exposure was minimized by prioritizing ICE for anatomical guidance and device deployment, resulting in limited fluoroscopy times despite the absence of TEE.

### Postprocedural management and follow-up

All patients received already aspirin before device implantation, due to preexisting stroke. All patients received the addition of clopidogrel 75mg from the device implantation, to obtain dual antiplatelet therapy for a minimum duration of 3 months.

A 12-lead electrocardiogram and TTE was obtained after the index procedure, allowing to check the correct position of the device and to ensure the absence of pericardial effusion, before hospital discharge.

During the in-hospital period, the following safety criteria were collected: periprocedural complications, including access-related complications (bleeding, false aneurysm, deep vein thrombosis, arteriovenous fistula), pericardial effusion, device migration, supraventricular tachycardia and vital status. Early migration or pericardial effusion was systematically checked on pre-discharge echocardiography. Supraventricular arrhythmias were detected during intraprocedural monitoring, on the discharge electrocardiogram, or in the presence of symptoms.

Vascular complications included bleeding, graded according to the Bleeding Academic Research Consortium (BARC) classification, pseudoaneurysm, deep vein thrombosis, and arteriovenous fistula. Doppler ultrasound was performed only in the presence of abnormal findings on pre-discharge clinical examination.

Patients were contacted by phone at 3 months. Data was collected regarding vital status, current treatments, cardiovascular events (ischemic stroke recurrence, myocardial infarction, heart failure hospitalization, atrial fibrillation), vascular complication, or any hemorrhagic event. A questionnaire was used to ensure a complete satisfaction of the patient and evaluate the feeling of the use of ICE catheter over TTE. Patient comfort was assessed by asking patients to rate their comfort from 0 (unbearable) to 10 (no discomfort) after the procedure.

### Study endpoints

Procedural success was defined as successful implantation of the PFO closure device with adequate stability and without device embolization. The presence of a moderate residual shunt immediately after the procedure was not considered procedural failure, as spontaneous resolution after device endothelialization may occur.

Study endpoints included early discharge defined as discharge within 24 h of admission, outpatient care defined as same-day discharge, patient-reported procedural comfort, and clinical outcomes assessed at three months.

Safety endpoints included in-hospital periprocedural complications such as access-related vascular complications, pericardial effusion requiring intervention, device migration, supraventricular arrhythmias, and all-cause mortality.

### Statistical analysis

Statistical analyses were performed using SPSS software (Statistical Package for the Social Sciences, version 12.0.1, IBM Inc, USA). Continuous variables were expressed as mean ± SD or median with interquartile range (IQR). Categorical variables were reported as counts and percentages.

### Ethics

This study was a retrospective observational analysis based exclusively on routinely collected clinical data and involved no additional intervention or modification of patient management. According to French public health law, such non-interventional retrospective studies are exempt from Institutional Review Board approval. This exemption is consistent with institutional policy at Dijon University Hospital. The study was conducted in accordance with the principles of the Declaration of Helsinki. All patients were informed that their anonymized clinical data could be used for research purposes, and no patient objected to such use. In addition, verbal consent was obtained during follow-up telephone contact for participation in the patient-reported outcome questionnaire and for publication of the results.

## Results

### Patients baseline characteristics

The baseline characteristics of patients are summarized in Table 1.

**Table 1:** Baseline characteristics of patients

*Demographic characteristics*	
Age (years)	46 ± 12
Male sex	119 (58)
Hypertension	27 (13.2)
Diabetes	6 (2.9)
Current smoker	51 (25)
Dyslipidemia	25 (12)
Body mass index ≥ 30	24 (12)
Estrogen-progestin contraceptives	19 (9.3)
Prior stroke	32 (15.6)
Prior MI	0 (0)
DVT or PE	18 (8.8)
*Qualifying event*	
Stroke	185 (90.2)
TIA	21 (10.2)
NIHSS	3.1 ± 5.1
ROPE Score	7.1 ± 1.6
CHA₂DS₂-VASc	1.0 ± 1.1
*Echocardiography*	
Left-intraventricular thrombus	0 (0)
Indexed LA volume (ml/m^2^)	22.5 ± 6.6
Atrial septal aneurysm	166 (82.6)
LVEF > 50%	201 (100)
Significant valvulopathy	2 (1.0)

Note: Data are presented as n (%) or Mean ± SD

DVT: deep veinous thrombosis, LA: left atrial, LVEF: left ventricular ejection fraction, MI: myocardial infarction, TIA: transient ischemic attack, PE: pulmonary embolism

The study included 205 patients with a mean age of 46 ± 12 years, and 58% were male. Among the female participants, 9.3% were using estrogen-progestin contraceptives. A history of prior ischemic stroke occurring before the qualifying cerebrovascular event leading to PFO closure was documented in 15.6% of patients.

The majority of patients presented with a stroke as the qualifying event (90%), while 10% had experienced a TIA. The average NIHSS Score was 3.1 ± 5.1, and the average ROPE Score, used to assess the likelihood that the stroke was related to PFO, was 7.1 ± 1.6. The CHA₂DS₂-VASc score, which estimates stroke risk in patients with atrial fibrillation, was low at 1.0 ± 1.1.

Echocardiographic evaluation revealed that 82.6% of patients had an ASA. The indexed left atrial volume was 22.5 ± 6.6 ml/m^2^, and all patients had a left ventricular ejection fraction > 50%, indicating preserved systolic function.

### Procedural features and efficacy

The data regarding the procedure are described in Table 2.

**Table 2:** Procedure characteristics and follow-up data of patients

*Procedure characteristics*	
Mean time AVC-PFO-closure (days)	437 ± 874
Mean time Multidisciplinary consultation meeting – PFO closure (days)	114 ± 362
Procedure under local anesthesia	205 (100)
Prosthesis size implanted	
- 25 mm	130 (63.4)
- 30 mm	33 (16.1)
- 35 mm	41 (20)
Closing system	
- PROGLIDE	130 (70.7)
- COMPRESSION	50 (27.2)
- BOTH	4 (2.2)
Mean procedure time (min)	56.5 ± 19.2
Mean Fluoroscopy time (min)	7.8 ± 5.4
X-ray dose (cGy.cm^2^)	1092 ± 2554
Success rate	205 (100)
Time to discharge	
- Outpatient care	91 (44.3)
- Within 24 h	193 (94.1)
*Complications*	
Vascular complication	7 (3.4)
Prosthesis embolization at discharge	0 (0)
Prosthesis embolization at 3 months	1 (0.5)
Atrial fibrillation or flutter	4 (2)
Acute coronary syndrome	1 (0.5)
Pericardial effusion	1 (0.5)
*Treatment 3 months after PFO-closure*	
Dual anti-platelet therapy	1 (0.5)
Single anti-platelet therapy	180 (87.8)
- Aspirin	163 (84)
- Clopidogrel	4 (2)
- Ticagrelor	1 (0.5)
Oral anticoagulation	16 (7.8)
- Apixaban	11 (5.4)
- Rivaroxaban	0 (0)
- Dabigatran	1 (0.5)
- VKA	4 (2)
*Telephone survey characteristics*	
Procedural comfort	9.1 ± 1.7 *(out of 10)*
Procedure-related anxiety	3.5 ± 3.3 *(out of 10)*

Note: Data are presented as n (%) or Mean ± SD

PFO closure was performed at a mean of 436 days following the stroke event and 114 days after the multidisciplinary consultation.

The percutaneous closure of PFO was successfully achieved in all 205 patients (100%). The procedure was performed under local anesthesia in all cases, with a mean procedure time of 56.5 ± 19.2 min and a mean fluoroscopy time of 7.8 ± 5.4 min. The mean radiation dose administered was 1092 ± 2554 cGy.cm^2^.

Prosthesis sizes of 25mm, 30mm, and 35mm were used, with the majority of patients receiving a 25mm device (63.41%). Vascular access closure was predominantly achieved using the Perclose ProGlide® system in 71% of patients.

Nearly all patients experienced early discharge, as 94% left the hospital within 24 h of admission, including 44% who underwent the procedure on an outpatient basis. Complications were rare, with vascular complications occurring in 3.4% of patients and no major bleeding events or other serious procedure-related complications. There were no cases of prosthesis embolization at discharge.

### Follow-up outcomes

At 3-month follow-up, the majority of patients (92%) were maintained on single antiplatelet therapy, with aspirin being the most common (84%). A minority required oral anticoagulation (7.8%), with apixaban used in 5.4% of patients.

No recurrent ischemic stroke or TIA was observed during follow-up, and no deaths were reported.

One case of late device embolization was identified. This occurred in a 26-year-old male patient with a PFO associated with a large ASA. A 35-mm Amplatzer™ Septal Occluder Cribriform device had been implanted under ICE guidance, with a minimal residual shunt noted at the end of the procedure. Follow-up TTE at 3 months revealed asymptomatic migration of the device to the left ventricular outflow tract, without significant hemodynamic obstruction (mean gradient 20 mmHg). The patient was anticoagulated and successfully treated by surgical device removal and direct PFO closure via minimally invasive thoracotomy.

A phone survey was conducted to assess patient-reported outcomes following PFO closure. The average comfort level during the procedure was rated as 9.1 ± 1.7 out of 10, indicating high procedural comfort. Procedure-related anxiety was reported as 3.5 ± 3.3 out of 10, reflecting generally low levels of anxiety associated with the procedure.

## Discussion

The adoption of transcatheter PFO closure as secondary prevention of PFO-related stroke has markedly increased over the past decade, supported by strong evidence and updated recommendations [18]. While TEE has long been the standard for procedural guidance due to its detailed anatomical visualization, its reliance on general anesthesia introduces patient discomfort, procedural delays, and the need for multidisciplinary coordination. In contrast, ICE offers a streamlined, operator-controlled imaging modality that allows the entire intervention to be performed under local anesthesia, within addition shorter procedural times and reduced fluoroscopy exposure compared to TEE-guided procedures, which translates to lower radiation doses for both patients and operators [19, 20].

Our study reinforces the growing evidence supporting ICE as a safe, efficient, and patient-friendly alternative to TEE for PFO closure. The 100% procedural success rate, absence of major complications at discharge, and excellent patient-reported comfort confirm that ICE guidance achieves comparable efficacy to TEE while substantially improving the procedural experience. These findings are consistent with prior studies demonstrating that PFO closure with ICE guidance is safe and efficiency [21, 22].

Previous studies have primarily focused on the technical feasibility of ICE-guided closure, comparisons between ICE and TEE, or specific access strategies. In contrast, the present study was designed to evaluate ICE as the cornerstone of a fully minimalist procedural and organizational strategy, extending beyond imaging guidance alone. The practical considerations described in the Methods section highlight key decision points when adopting a minimalist ICE-guided strategy. The key originality of our work lies in the systematic integration of ICE into a simplified workflow combining local anesthesia, limited vascular access, and early discharge. In a relatively large, consecutive, single-center cohort, we demonstrate that this approach is not only feasible and safe, but also reproducible in routine clinical practice. Procedural success was achieved in all patients, with short procedure times and a very low complication rate, confirming that procedural simplification does not compromise safety or efficacy.

Importantly, our study moves beyond traditional technical endpoints by incorporating patient-reported outcomes, which remain underrepresented in prior ICE-guided PFO closure series. The high comfort and satisfaction scores observed in our population (mean 9.1/10) and the low stress level (mean 3.5/10) underline a central advantage of this approach. Avoiding general anesthesia and esophageal instrumentation minimizes anxiety, accelerates recovery, and allows same-day discharge in the majority of cases. This contributes to redefining PFO closure not only as an interventional procedure but as part of a comprehensive patient-centered care pathway. In our cohort, procedural times were short (mean duration < 1 h from catheterization laboratory entry to exit), and nearly all of the patients were discharged within 24 h, including 44% on an outpatient basis. The consistently early discharge observed in our cohort further distinguishes this study from earlier reports and this ambulatory strategy represents an important evolution beyond earlier ICE-guided studies, which largely focused on intraprocedural metrics rather than post-procedural organization of care. These results support the feasibility of a fully ambulatory strategy, consistent with a previous meta-analysis of the literature conducted with a total of 11 studies, involving 4748 patients, and reporting shorter procedures, reduced radiation exposure, and decreased hospitalization time with ICE compared to TEE [14]. By removing the need for anesthesiology teams and TEE operators, this minimalist strategy may also reduce resource utilization and procedural delays, particularly in high-volume centers.

Our study confirms that ICE offers similar anatomical visualization to TEE while allowing for the procedure to be performed under local anesthesia, thus enhancing patient comfort and reducing the need for general anesthesia. Furthermore, in the literature, ICE could offer superior imaging of the fossa ovalis and the atrial septum due to its proximity to the PFO, facilitating precise device placement and monitoring throughout the procedure. This enhanced visualization potentially improves procedural outcomes and provides real-time guidance that is more direct than TEE [23, 24]. Nevertheless, validation of ICE images remains a consideration, especially in patients with complex anatomical variations, such as those with an atrial septal aneurysm. Vigna et al. [25] reported discrepancies between preprocedural TEE and intraprocedural ICE images, although the clinical significance of these differences is not fully understood. This highlights the need for careful preprocedural evaluation and integration of all imaging modalities in complex cases.

Complication rates were low. Minor vascular events occurred in 3.4% of patients, and only one case of device embolization was noted during follow-up. Although uncommon, this complication highlights the importance of careful intraprocedural stability assessment and close follow-up in patients with complex septal anatomy. Atrial fibrillation or flutter occurred in 2% of patients, lower than the incidence reported in previous studies but showing that arrhythmic complications should be considered in post-procedural monitoring [26, 27]. At 3 months’ follow-up, no recurrent ischemic events were observed. These data align with previously published series, where ICE-guided PFO closure demonstrated excellent safety and mid-term outcomes [28, 29].

Our study has limitations. The retrospective design and absence of a TEE-guided control group preclude direct comparative conclusions. Indeed, the procedural learning curve may have influenced outcomes. Despite these limitations, these findings support ICE as a cornerstone imaging modality for the next generation of minimalist structural heart interventions, aligning safety, efficacy, and patient-centered care. Thus, our results support its role as a central enabler of a minimalist, ambulatory, and patient-centered PFO closure strategy.

## Conclusion

ICE–guided PFO closure performed under local anesthesia is a safe, effective, and well-tolerated procedure that supports a fully ambulatory strategy. In our experience, procedural success was universal, complication rates were low, and patient satisfaction was high. By eliminating the need for general anesthesia and transesophageal guidance, this minimalist strategy simplifies logistics, optimizes resource use, and enhances the overall procedural experience. These findings support ICE as a central imaging modality for next-generation structural interventions, bridging technical efficacy and patient-centered care.

## Data Availability

The data that support the findings of this study are available from the corresponding author, Dr. Thibaut Pommier, upon reasonable request. Access to de-identified data will be granted for academic and non-commercial research purposes, subject to approval by the local ethics committee and a data-sharing agreement.

## Abbreviations

PFO: Patent foramen ovale
TEE: Transesophageal echocardiography
ICE: Intracardiac echocardiography
TIA: Transient ischemic attack
TTE: Transthoracic echocardiography
ESUS: Embolic stroke of undetermined source
ASA: Atrial septal aneurysm
ASD: Atrial septal defect
